# Instagram, Flickr, or Twitter: Assessing the usability of social media data for visitor monitoring in protected areas

**DOI:** 10.1038/s41598-017-18007-4

**Published:** 2017-12-14

**Authors:** Henrikki Tenkanen, Enrico Di Minin, Vuokko Heikinheimo, Anna Hausmann, Marna Herbst, Liisa Kajala, Tuuli Toivonen

**Affiliations:** 1 Digital Geography Lab, Department of Geosciences & Geography, University of Helsinki, Helsinki, FI-00014 Finland; 2 School of Life Sciences, University of KwaZulu-Natal, Durban, 4041 South Africa; 3 South African National Parks, Scientific Services, Phalaborwa, 1390 South Africa; 4 Metsähallitus, Luontopalvelut, Savonlinna, FI-57130 Finland

## Abstract

Social media data is increasingly used as a proxy for human activity in different environments, including protected areas, where collecting visitor information is often laborious and expensive, but important for management and marketing. Here, we compared data from Instagram, Twitter and Flickr, and assessed systematically how park popularity and temporal visitor counts derived from social media data perform against high-precision visitor statistics in 56 national parks in Finland and South Africa in 2014. We show that social media activity is highly associated with park popularity, and social media-based monthly visitation patterns match relatively well with the official visitor counts. However, there were considerable differences between platforms as Instagram clearly outperformed Twitter and Flickr. Furthermore, we show that social media data tend to perform better in more visited parks, and should always be used with caution. Based on stakeholder discussions we identified potential reasons why social media data and visitor statistics might not match: the geography and profile of the park, the visitor profile, and sudden events. Overall the results are encouraging in broader terms: Over 60% of the national parks globally have Twitter or Instagram activity, which could potentially inform global nature conservation.

## Introduction

Tourism is one of the largest industries in the world, generating more than US$ 1.5 trillion annually and contributing up to 10% of the world’s GDP^1^. Recreation and nature-based tourism are key sectors for tourism, and protected areas are the cornerstone of these industries, receiving approximately 8 billion visitors a year^2^. Protected areas, such as national parks, help meet the local and international biodiversity targets^3^. They often have high recreational value and hence are significant for national and local economies^4^. Protected areas are also important for providing people access to benefits from cultural ecosystem services^5^.

Understanding the number of visitors in national parks and other natural areas is essential for their management and marketing^6^. An increase in tourists’ number may result in a higher disturbance on biodiversity (e.g. trampling on plants^7^, disruption of feeding and breeding^8,9^, and decrease in species reproductive success^10^) and pressure on the environment (e.g. resource consumption, pollution, erosion, habitat loss^11^), challenging the sustainability of tourism^12^. Visitor monitoring can help to allocate resources, target infrastructure development, and restrict access to areas where human pressure is unsustainable^13^, hence minimizing the impact on the biodiversity. Monitoring visitor rates is also crucial for assessing the recreational value of national parks and other green areas^14,15^. Evaluating and demonstrating the benefits that recreation brings to the local economy and human well-being, compared to other land uses^16^, is often of paramount importance in justifying the existence of protected areas. However, obtaining up-to-date information about how people use and visit natural areas is often laborious, time-consuming and costly by traditional means such as surveys, interviews, GPS-trackers, or counters^17,18^. As a result, researchers have started increasingly to address this limitation^18,19^ by exploiting ‘big data’, i.e. large quantities of data continuously generated by the ubiquitous digital devices.

Big data have been previously used to understand various aspects of human behaviour such as peoples’ localities and mobilities in different environments^20–22^, including estimating the number of visitors to recreational and protected areas^18,19,23,24^. In the big data domain, social media platforms are a particularly promising source of information because i) the data is often openly available free of charge; ii) it is relatively easy to collect; iii) it has good spatial coverage at multiple scales (from local to global level); iv) it is generated continuously; v) and it is rich in content^17,22,25–27^.

There is a wide selection of popular social media platforms. The most popular platform in the world, Facebook, is difficult to use for extensive research as access to data is limited. Hence, scientists have used other platforms providing more easy access to public data. Among these, Flickr is one of the oldest social media platforms (established in 2004), popular especially for sharing pictures, whereas Twitter (established in 2006) is arguably the most used short-text discussion forum in the world. Newer platforms such as Instagram (established in 2010) have recently gained popularity among people owning smartphones with high-quality cameras. Out of these platforms, Twitter has been extensively used in research in different fields^28,29^, whereas Flickr, and Panoramio (service was closed in 2016), have been used more in the environmental sciences^18,19,26,30^. Instagram remains a relatively unexplored source of data for research, although it has been gaining momentum recently^17,22,25,26,31^.

Thus far, most studies utilising social media data for analysing recreational use have relied on single social media platforms^19,23,24^, or used social media data along with other type of crowdsourced information (Wikipedia, OpenStreetMap)^32^. In addition, only few studies have used more controlled data sources, such as surveys, official statistics and interviews, to validate the use of social media data as a proxy for visitation, and popularity, across different natural and cultural attractions^19,23,24,32–34^. A systematic assessment of the usability of different social media platforms in relation to visitation in protected areas such as national parks is, hence, lacking.

**Figure 1:**
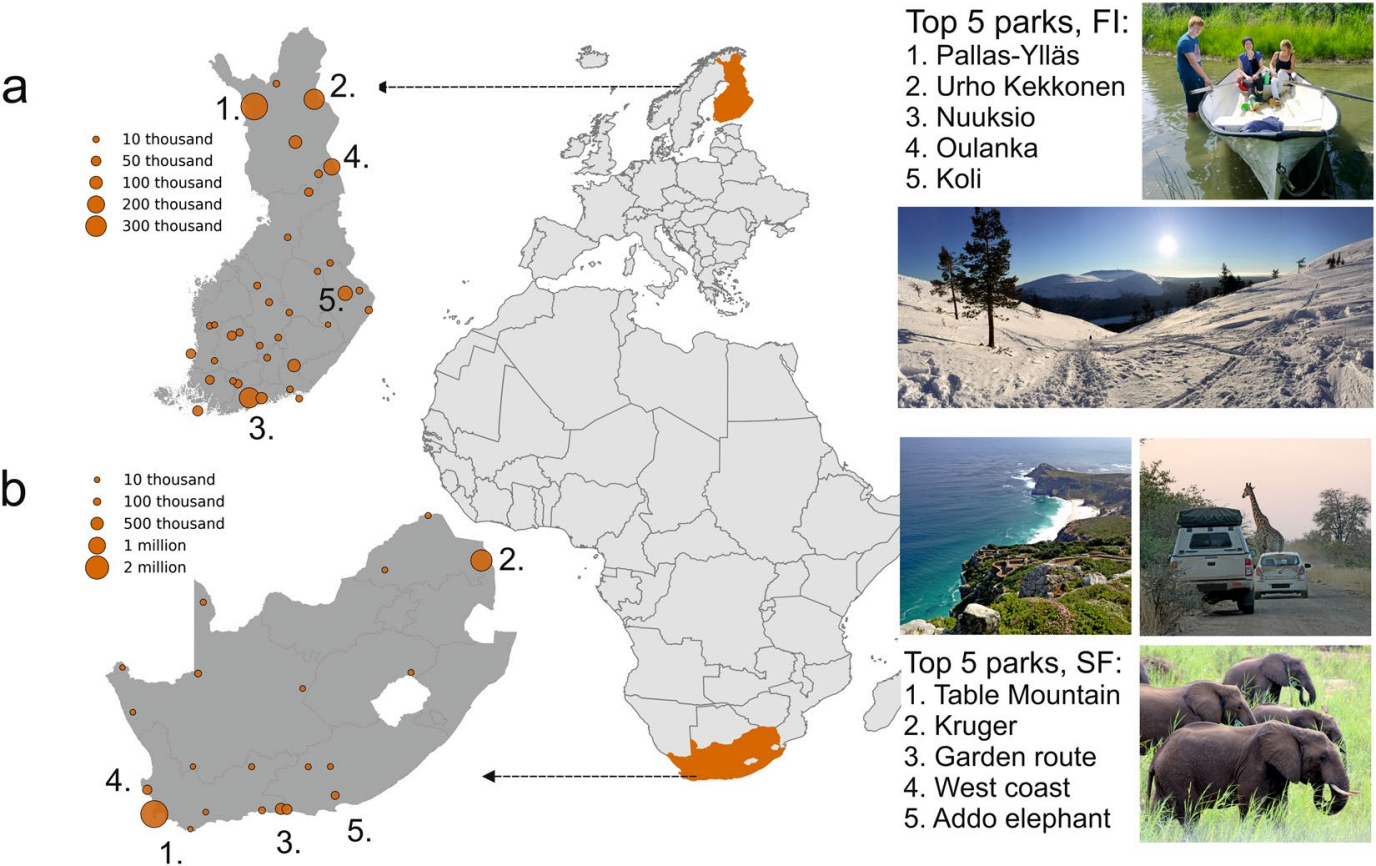
The locations of the national parks in Finland (**a**) and South Africa (**b**) where the size of the circle indicates the number of tourists visiting the given park in 2014. Numbered locations show the five most visited national parks in Finland and in South Africa. The photos show examples of the differences in the type of nature-based tourism between the two countries: a more charismatic wildlife and landscape viewing focus in South Africa, and a higher variety (e.g. landscape seasonality, activities) of attractions in Finland^15^. Photo credits: Anna Hausmann, Tuuli Toivonen, Henrikki Tenkanen. Figure has been created with Matplotlib v2.02^62^, Geoplot v0.0.3 and Geopandas v0.2.1 modules in Python 3.5.3 programming language (https://www.python.org/) under the PSF License (docs.python.org/3/license.html) using openly available World Borders Dataset (unmodified) from http://thematicmapping.org/downloads/world_borders.php under Creative Commons BY-SA licence (https://creativecommons.org/licenses/by-sa/3.0/).

In this paper, we analyse social media data collected from three platforms that are mostly used for research purposes, namely Instagram, Twitter and Flickr, and assess how well these data, separately and combined, can be used to estimate visitation patterns in national parks. In particular, we systematically investigate whether or not social media data can identify 1) the popularity of the parks (ranking), and 2) the monthly visitation rates of the parks. Furthermore, we 3) assess whether there are platform-specific differences in the correlations between social media data and official visitor statistics. In addition to statistical comparisons, we 4) identify different factors that could explain where social media data has a strong relationship with official visitor statistics, and where it does not. We do this by conducting semi-structured group-interviews with stakeholders, capturing their interpretations of these results. We conduct our analyses in 56 national parks located in two different countries: 21 in South Africa and 35 in Finland (Fig. 1a,b). The chosen countries form a good sample for the study as they differ in culture, economy, biodiversity, climate, and tourism profiles. In addition, South African and Finnish park authorities both collect up-to-date official visitor statistics that are needed to address the study questions.

## Results

### Park popularity

**Figure 2:**
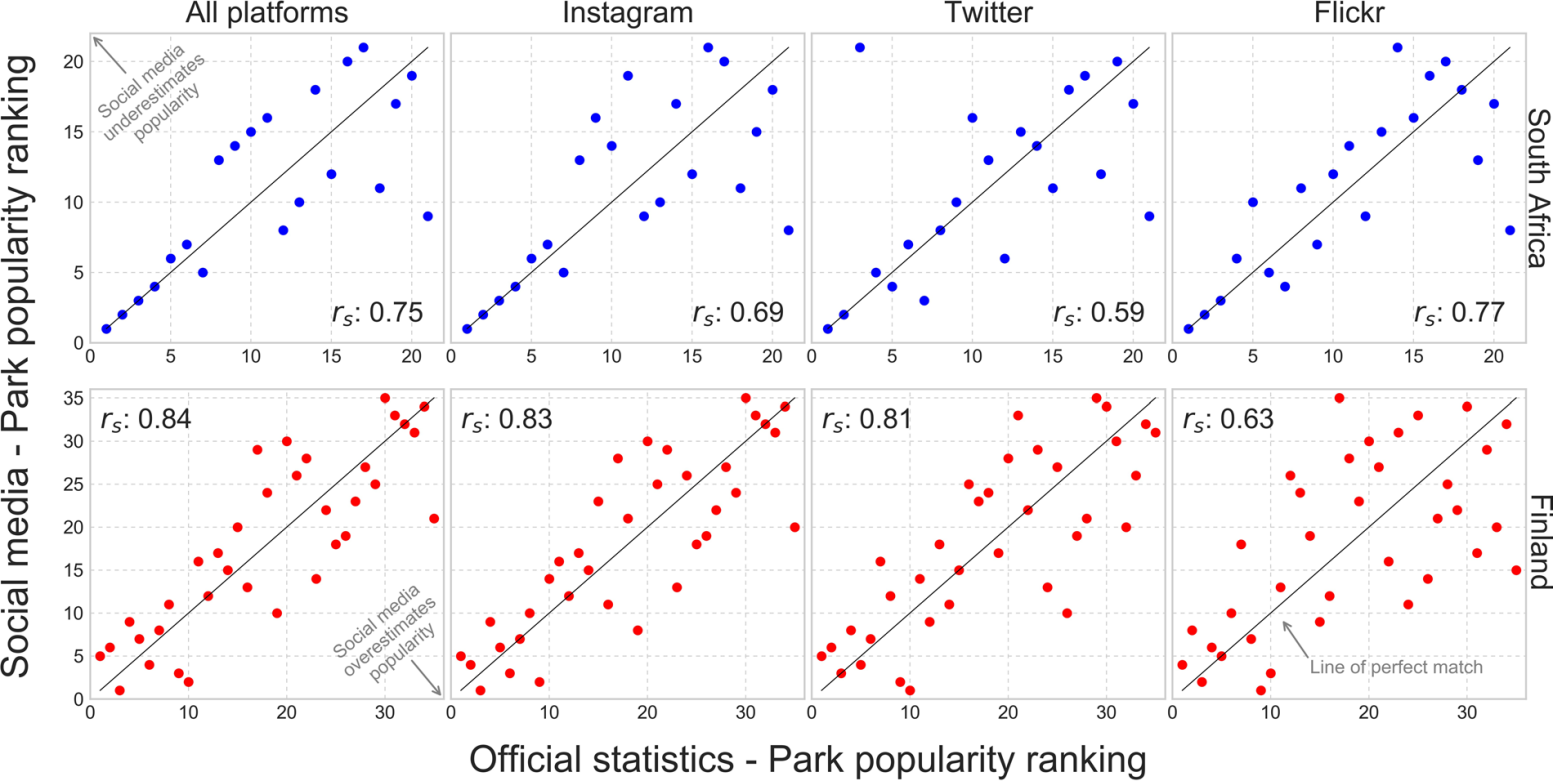
Park popularity comparisons between official visitors and social media data from different platforms reveal that in general social media data matches well with the park popularity having Spearman’s rank correlation 0.75 in South Africa and 0.84 in Finland. There are differences between platforms: e.g. in South Africa Instagram predicts the 4 most popular parks correctly whereas Twitter only 2. Value 1 corresponds to the most visited park based on official statistics (x-axis) and social media (y-axis), and values 21 (South-Africa) and 35 (Finland) correspond to the least visited park accordingly. Figure has been created with Matplotlib v2.02^62^ and Pandas v0.19.2 modules in Python 3.5.3 programming language (https://www.python.org/) under the PSF License (docs.python.org/3/license.html).

Comparison between social media posts and official visitor statistics show that all social media platforms can relatively well reveal the popularity of the parks. This means that officially more visited parks are more popular also in social media and vice versa (Fig. 2). This applies to both of our study countries, although in Finland where there are more parks, the Spearman’s rank order correlations (r_s_) are higher than in South Africa. The popularity ranking of the parks works particularly well for the most visited parks. For less popular parks, the ranking becomes less accurate. For instance in South Africa, Instagram reveals correctly the park popularity rank order of the 4 biggest national parks, but tends to over or underestimate the popularity of the less visited parks. While all platforms provide meaningful information on the park popularity, there are interesting differences between platforms. In Finland, Instagram worked best for estimating park popularity. In South Africa, on the other hand, the least popular platform Flickr (see Table 1 in Methods section) provided the highest correlation with the official statistics (Fig. 2). In Finland Flickr had the lowest rank correlation.

**Table 1:** Statistics about South African and Finnish national park official visitors and statistics about social media data posted from the countries.

Statistic	South Africa	Finland	Total
**Official visitors**	**5 855 336**	**2 177 142**	**8 032 478**
**Social media posts**	**61 264**	**21 933**	**83 197**
Instagram	29 251	9 082	38 333
Twitter	20 974	11 473	32 447
Flickr	11 039	1 378	12 417
**Unique social media users**	**13 633**	**5 662**	**19 295**
Instagram	10 066	4 627	14 693
Twitter	3 100	902	4 002
Flickr	467	133	600
**Social media user days**	**24 886**	**8 456**	**33 342**
Instagram	17 728	5 873	23 601
Twitter	5 880	2 363	8 243
Flickr	1 278	220	1 498

### Temporal visitation patterns

When extending the inspection to temporal visitation pattern, i.e. the monthly visitation rate of each park, our results show that the visitation patterns derived from social media follow relatively well the visitation patterns seen in the official statistics. In 10% of the parks, the temporal patterns between social media and official statistics have nearly perfect match (Pearson’s correlation > 0.9). Half of the parks (28/56) have equal or higher than 0.7 Pearson’s correlation between the official monthly visitor count and the social media user-days of the respective month. 64% (36/56) of the parks have equal or higher than 0.6 correlation. On the other hand, 20% of the parks (11/56) have correlations below 0.3 and five of them have negative correlations where patterns do not match.

**Figure 3:**
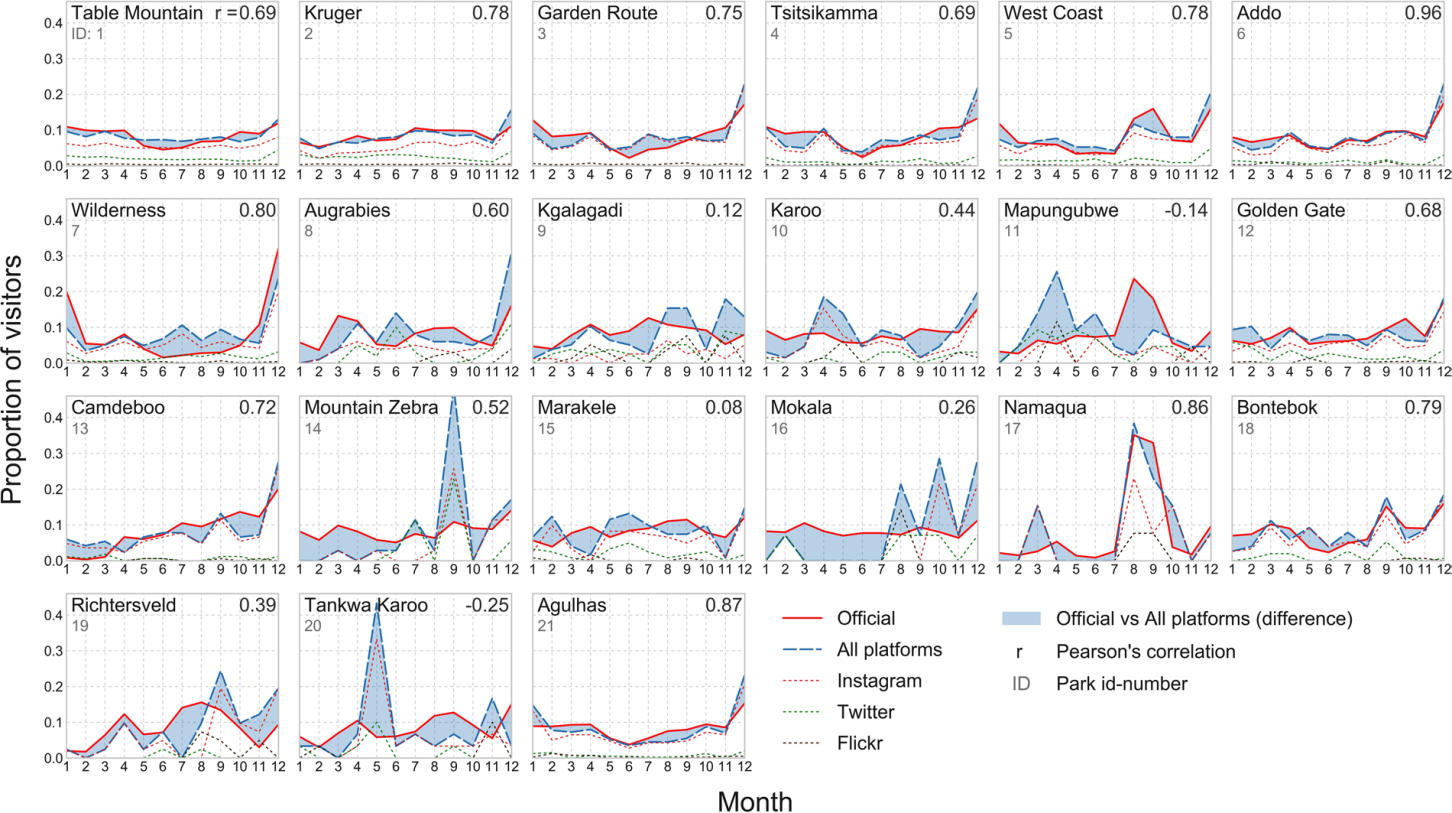
Comparison between official visitor statistics and social media data in 21 national parks in South Africa. The lines of the individual platforms show that Flickr is always the least significant in terms of volume of the data, whereas Instagram is often the most data-rich platform. Figure has been created with Matplotlib v2.02^62^ and Pandas v0.19.2 modules in Python 3.5.3 programming language (https://www.python.org/) under the PSF License (docs.python.org/3/license.html).

**Figure 4:**
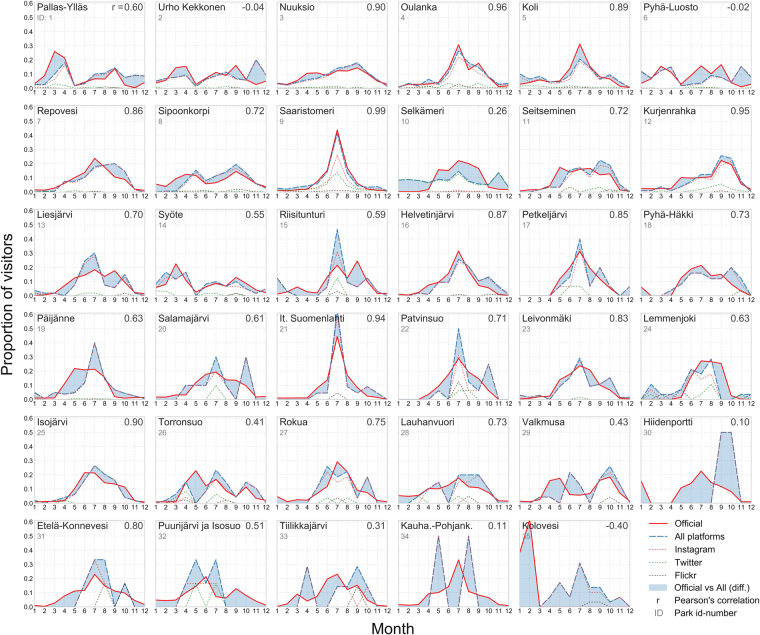
Comparison between official visitor statistics and social media data in 35 national parks in Finland. The plots (in Figs 3 and 4) are in a descending order based on the number of visitors in the parks. Plots reveal that social media data tend to perform in a robust manner in the more visited parks, whereas in the least popular parks the patterns differ significantly. Figure has been created with Matplotlib v2.02^62^ and Pandas v0.19.2 modules in Python 3.5 programming language (https://www.python.org/) under the PSF License (docs.python.org/3/license.html).

Figures 3 and 4 visualize the similarity of the monthly variation between official visitor statistics and social media data. The curves show the monthly share of tourists (full year equals to 100%) visiting national parks in 2014 in South Africa (n = 21) and in Finland (n = 35), and the temporal variation of data from three social media platforms (Instagram, Twitter and Flickr) separately and using their combination (sum of all platforms). The social media data is reported in the number of “social media user-days” (SUD), i.e. the sum of days a user has been active in a park (see Methods), instead of raw post counts. We found slight temporal autocorrelation with one or two lags (with 95% confidence interval) in some of the parks (3/21 national parks in South Africa, and 17/35 in Finland). In these parks, the correlation coefficients should be considered with caution, as the estimates might be inflated due to temporal autocorrelation (see Supplement S1 for details). Hence, we excluded parks with temporal autocorrelation from further analyses.

### Differences between platforms

**Figure 5:**
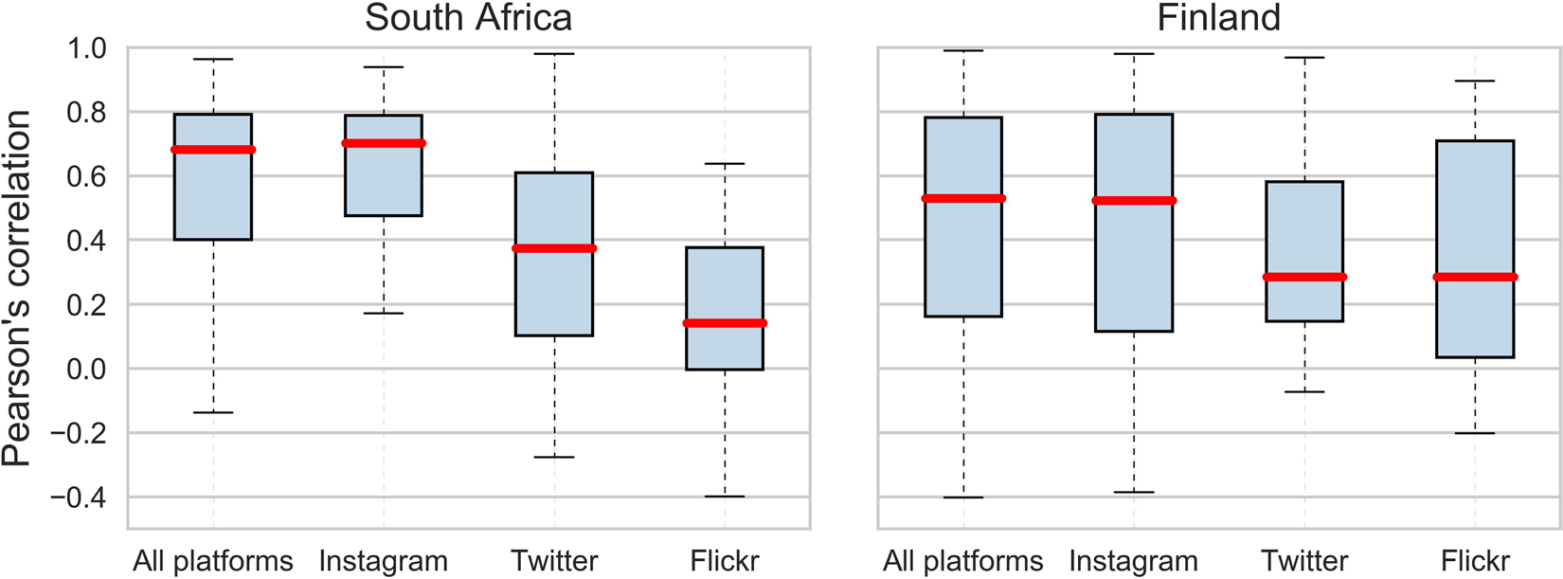
Boxplots reveal that Instagram performs best in estimating the monthly visitors when measured with Pearson’s correlation coefficients between official visitor statistics and social media user-days. The performance of Instagram is slightly better and more robust in South Africa than in Finland, having a 70% median correlation. This figure is based on parks (N = 36) where the data is not temporally autocorrelated. Result with all parks is presented in Supplement S2. Figure has been created with Matplotlib v2.02^62^ and Pandas v0.19.2 modules in Python 3.5.3 programming language (https://www.python.org/) under the PSF License (docs.python.org/3/license.html).

The results reveal that Instagram outperforms Twitter and Flickr in representing the monthly visitor patterns. In South Africa, Instagram has a median correlation of 0.7, which is more than twice as high as the median correlation of Twitter and Flickr median (Fig. 5). Furthermore, the variance of the correlations is smallest with Instagram, also highlighting that the relationship between official visitation rates and Instagram is more robust than with Twitter and Flickr. In Finland, Instagram also performs better than Twitter and Flickr, but the differences are less evident than in South Africa. Kruskal-Wallis test confirms that the differences between groups (park-wise correlations with Instagram, Flickr and Twitter) are statistically significant in South Africa (p-value: 0.007), whereas in Finland they are not (p-value: 0.55). Furthermore, Dunn’s post hoc test with adjusted p-values (Holm-Sidak) reveal that the difference is significant between Instagram and Twitter (p-value: 0.02) and between Instagram and Flickr (p-value: 0.0002). When including all parks (also the ones with temporal autocorrelation) the differences between platforms are statistically significant also in Finland (see Supplement S2).

### Influence of park popularity

**Figure 6:**
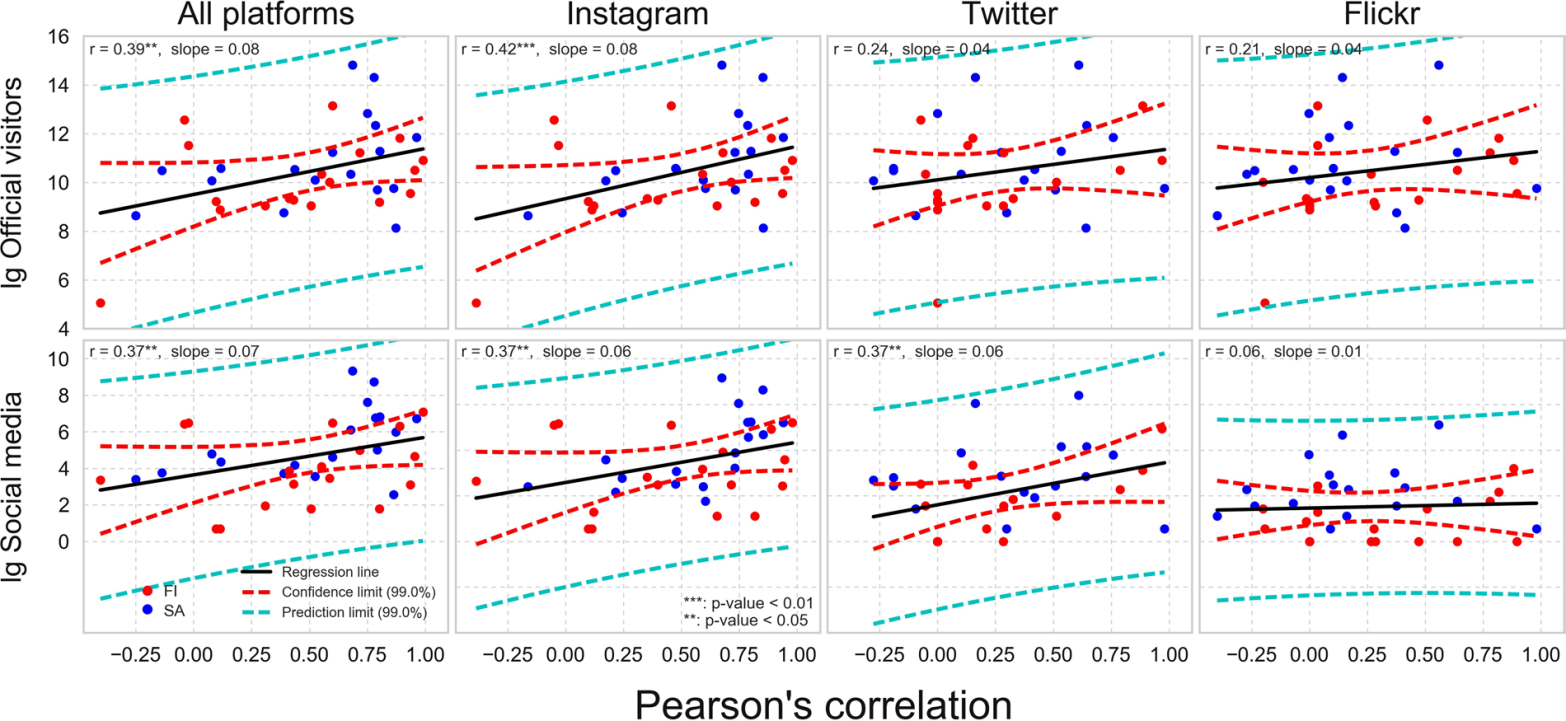
Social media data tend to work better in more visited parks (top row) and in parks having higher number of social media user-days (bottom row) which is shown here by comparing the Pearson correlation coefficients (between social media and official visitor statistics, see Figs 3 and 4) against log-transformed number of official visitors and social media user-days. This trend is visible particularly with Instagram data and all platforms combined, and weaker or non-existing with Twitter and Flickr. This figure is based on parks (N = 36) where the data is not temporally autocorrelated. Result with all parks is presented in Supplement S2. Figure has been created with Matplotlib v2.02^62^ and Pandas v0.19.2 modules in Python 3.5 programming language (www.python.org/) under the PSF License (docs.python.org/3/license.html).

The match between the official monthly visitor rates and the social media user-days seems to be higher in more popular parks, compared to less popular ones (see Figs 3 and 4), although there are clear exceptions like Urho Kekkonen in Finland and Agulhas in South Africa. In Fig. 6 we investigate this relationship further and confirm that social media data tends to match with the monthly visitation patterns better in more visited parks (higher visitor numbers) and in parks having higher quantity of social media content (and users), although the relationship is fairly weak. The results show ascending trend lines especially for the combination of all platforms and Instagram (with highest Pearson correlations and slope). The more scattered patterns from Twitter and Flickr reveal that those platforms tend to work more unevenly regardless of the park popularity.

### Expert interpretations of the results

**Figure 7:**
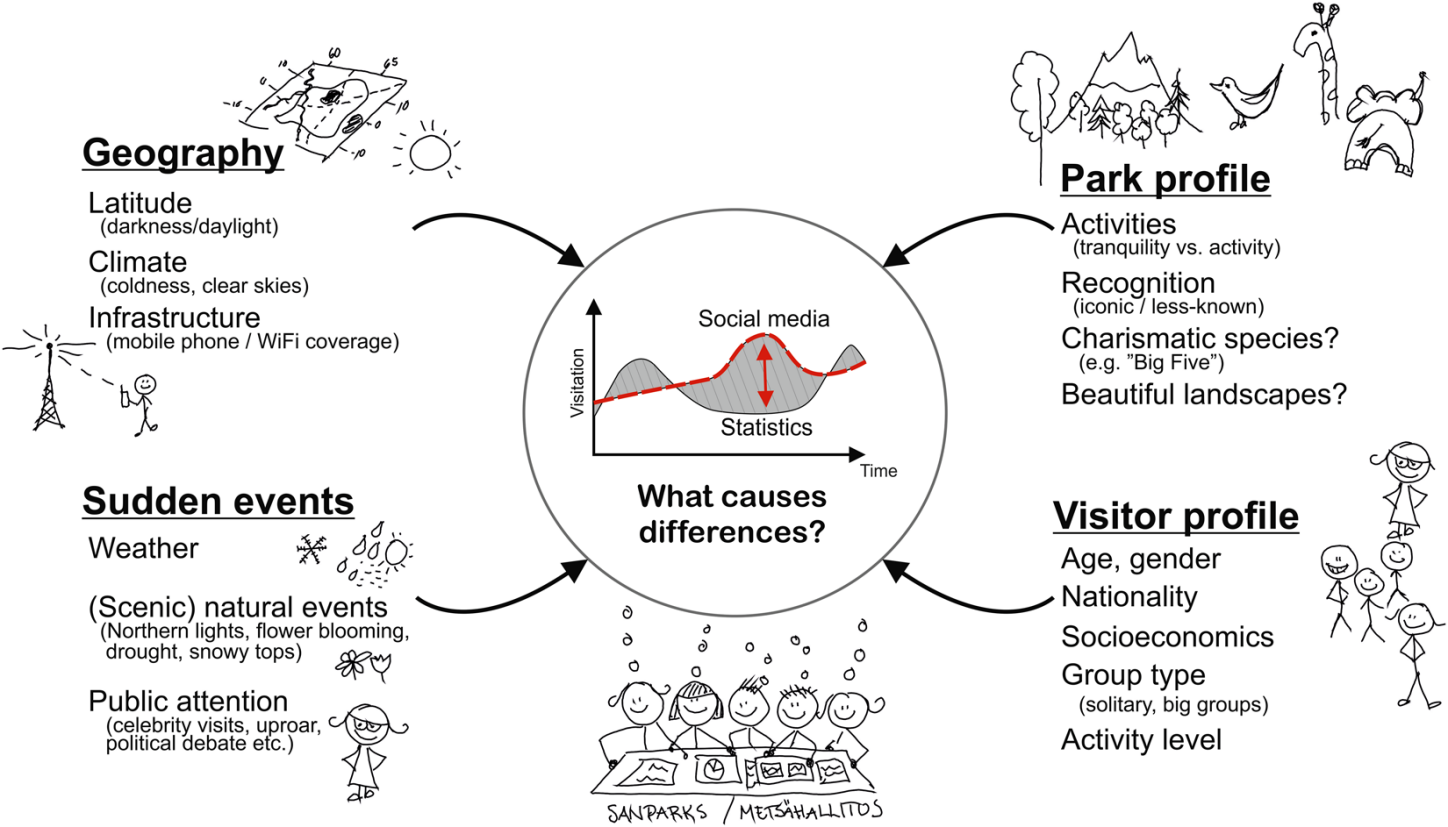
Conceptualisation of the potential reasons explaining the differences between the social media user-days and the official visitor statistics. We identified a number of park-specific reasons in stakeholder workshops (one in Finland and two in South Africa). Here we classified them into four main categories that could help to explain why the patterns derived from social media does not always match well the official visitor statistics. Figure was created in CorelDRAW Graphics Suite version X8 (www.coreldraw.com/en/). Illustrations were drawn by Tuuli Toivonen.

In order to better understand our results, we organised stakeholder meetings in South Africa and in Finland with the park managers from the respective countries. The park managers were asked to identify reasons that could potentially explain the differences in social media and the official visitor statistics in each park. We grouped their suggestions into four broad categories (see Figs 7 and Supplement S3). Firstly, the geography and location of the park was considered as a likely explanation: in areas of high latitudes, for example, the winter darkness and coldness influences the technical possibilities to take good photos outdoors, which may lower the number of social media posts in comparison to visitor numbers during the winter months. Secondly, the park profile is important: some parks attract visitors with quietness and others with adventurous activities. Parks with activities might get more social media usage overall, and if the profile varies between seasons this could cause differences in the curves (e.g. seeking for quietness in winter, adventurous activities in summer). Thirdly, visitor profile is important: there are clear age and gender differences in the use of social media, skewed towards younger people and women posting more^25,31,35^. Hence, parks that offer activities for younger people tend to have higher relative number of posts and for example the school holiday periods might be overrepresented in the social media data. Fourthly and finally, sudden events may decrease or increase the relative number of social media posts: rain and poor weather make it difficult to take photos, while a particularly beautiful spring flower blooming or a celebrity visit may increase their relative amount.

## Discussion

Our results show that social media data has a high potential to be used as a data source for monitoring the number of visitors in natural areas, both for gaining information on the park popularity and on temporal visitation patterns. The results match the findings of earlier studies that have estimated the popularity (ranking) of protected areas and other touristic attractions^24,32,36,37^, and their temporal visitation patterns^18,24^, but mostly focusing on a single social media platform. Our systematic analysis highlights the need for using data from multiple sources and calls for caution when interpreting the results.

Different types of crowdsourced data vary in quality, information and characterization, together with the ability of predicting visitation rates^32^. Sessions *et al*.^18^ have earlier reported positive results in predicting monthly visitor patterns in national parks with Flickr data aggregated from multiple years. Our results demonstrate the usefulness of social media data even in single year analyses, if the platform is popular enough. With Flickr, for example, the amount of observations per year is too low in many national parks for it to perform adequately with data only from a single year. In our study areas, the correlation between Instagram and the monthly visitation rates were up to twice as strong as when comparing to the other two platforms. This is important as one of the main assets of using continuous social media data is to observe changes in the visitation patterns which might get hidden when aggregating data over the years.

When comparing the performance of different social media platforms, we found that Instagram provides most consistently successful results in single platform analyses. However, all three platforms combined yield the most robust matches between the official visitor statistics and social media data. On one hand, this is likely due to having access to higher number of posts per area from larger number of social media users. On the other hand, we suggest that platforms are used differently according the users’ needs or behavior: they can be used to share every-day activities and experiences (Instagram), thoughts and ideas (Twitter), and/or high-quality professional photographs (Flickr)^38^. Using all these data sources together increases the breadth of the data, which results in more robust results.

Although social media data follow relatively well visitor patterns from the official statistics, we show that caution is needed when using it as a surrogate of visitor counts^18,19^. Social media data tend to work better in more visited parks and places where more social media content has been uploaded. This pattern is not, however, consistent. Our results contain unvisited small parks that have high correlations but also highly visited parks with significant discrepancy between the patterns of social media and statistics. Hence, the estimates of park popularity and temporal patterns from social media data should always be taken with caution and, if possible, evaluated against other data sources. When doing so, it must also be noted that even official visitor statistics are not perfect. For instance, in Finland all the parks can be accessed without any official registration. Hence, the visitors are mostly estimated with automatic counters along the footpaths. In South Africa, most of the parks are entered via gates and the visitation numbers are mostly based on entrance tickets or registration forms making them fairly reliable. Not all parks, however, have such a system (e.g. Table Mountain national park which is accessible also without buying a ticket). In some areas, surveys are used to estimate the visitation numbers (e.g. in the Finnish archipelago area) that are prone to sampling bias in a similar manner as social media has age and gender bias^35^. Furthermore, certain areas lack totally the information about the number of visitors. In such places expert opinions could be particularly useful to evaluate the robustness of the results.

It is also important to critically assess different biases related to the social media platforms^39,40^. For instance, earlier survey based studies in Finland^22^ and South Africa^25^ have found that social media users in national parks tend to be younger than average visitors and that usage decreases significantly with age. Social media data also tend to contain noise caused by bots^41^ which might produce irrelevant content. Also inaccuracies in spatial location of the posts^42^ might cause irrelevant posts inside the parks. However, in Finnish and South African national parks earlier studies^22,25^ have shown that more than 95% of the social media content posted is related to the actual visit. One of the challenges related to social media data is the participation inequality, meaning that a small fraction of people produces most of the content^43,44^. To overcome this bias, one of the solution is to take into account only one post per day per user instead of using the total number of posts when using social media as a surrogate for visitor numbers^18,19,23,24^, as we did in this study.

It is also crucial to keep in mind that the usability of social media data as a proxy for visitation depends on the temporal granularity. Our results show that on a monthly basis, social media performs relatively well. However, making observations on daily visitor patterns would be challenging due to lack of observations from social media. Furthermore, the temporal granularity of the data plays a role in terms of the availability of methods: monthly level data from a single year limits the possibility to use more sophisticated statistical methods such as regression models^18^ with predictive power (e.g. GLM, GLS, ARIMA) due to low number of observations (n = 12).

We argue that with data fusion it is possible to gain more comprehensive understanding about a studied phenomenon when using social media data. By combining data from different sources, it is not only possible to gain more observations and robustness to the results, but also highly important because people are using multiple platforms simultaneously for multimodal communication including e.g. photos, text and videos^45^. Therefore, instead of big data revolution, we should aim at ‘all data revolution’^46^ considering all different data sources available and evaluating them with expert views. When doing so, biases and ethical questions related to social media data should also be considered carefully^46–51^. The research community should also be active in making data acquisition procedures clearer. As popular platforms are operated by private companies whose policies may change, the terms of service and access to the data might be blocked or get limited. For instance, Instagram used to have an open access to their API for public posts, but nowadays the access is limited mostly to commercial purposes (advertisers, broadcasters etc.) making it more complicated platform to use in research purposes. Therefore, it is important to advance efforts where social media data becomes archived for research use, and to have methods to share data responsibly^51–53^.

Our study combined data from two very distinct countries, with different profiles of national park visitors. In South Africa, the number of foreign visitors in national parks is considerably higher than in Finland, the cultural background is much more varied and people use the parks differently. For example, in Finland most national parks are visited for outdoor activities such as hiking or skiing, while in South Africa game driving and observing large-bodied charismatic species are the most popular attractions^54^. Regardless of the country-wise differences, our results are robustly suggesting that social media data has the potential to inform about visitor patterns in national parks. This is encouraging also for other areas of the world, where social media platforms are being used, but no official statistics are similarly available as in our example countries. Social media data is also available across country borders and could inform global conservation^55^. For example, it could contribute to estimating the global scale visitation patterns in protected areas in a similar manner as was done by Balmford *et al*.^2^ using visitor statistics. Moreover, social media data could be used to assess the intensity of human activities at a global scale, in order to inform spatial conservation prioritization of protected areas under pressure^3^. While we found that more than half of the national parks worldwide have social media activity, country-wise differences in social media platform popularity may be a limitation to this result. Hence, including platforms such as QQ or Sina Weibo (popular among Chinese speaking population) or VKontakte (popular in the Russian speaking world), may help improve the coverage of underrepresented countries.

## Materials and Methods

### Study area

The study focuses on two countries, South Africa and Finland, which received respectively 9.5 million and 2.7 million of international tourist arrivals in 2014. In both countries, tourism is an important part of the economy, generating a revenue of US$ 9.3 billion in South Africa and US$ 3.6 billion in Finland. Nature-based tourism in protected areas is in high demand among both national and international tourists, and key in producing financial, political and social support to protected areas^56^. However, these countries differ substantially as nature-based tourism destinations and attract a variety of tourist markets.

South Africa is a well-known wildlife-viewing destination, attracting markets of national and international tourists interested in charismatic megafauna, such as the “Big Five”^54^. However, South Africa´s national parks cover a large variety of biomes with different characteristics which attract different markets of tourists^57^ and explain visitor´s numbers in each site^58^. On the other hand, Finland´s national parks provide tourists with a “Nordic wilderness” experience^59^, which has smaller wildlife-viewing component. Opportunities for recreational activities, such as skiing, hiking, camping, fishing, are among the main factors explaining visitor´s numbers in Finnish national parks^15,60^.

### Official visitor statistics

We had access to official visitor statistics from 21 national parks in South Africa and 35 parks in Finland for year 2014 via national park authorities (SANParks in South Africa, Metsähallitus in Finland). According to the official statistics (see Table 1), there were in total around 5.9 million tourists visiting the national parks in South Africa, and 2.2 million in Finland. In South Africa, the visitor numbers are typically based on the data about entrance tickets since visitors need to register and buy a ticket before accessing national parks. In Finland, the visitor numbers are typically based on automatic electronic counters that have been installed along popular pathways inside the parks. Local correction factors are applied to counter data in order to account for known errors in counter readings. In addition, estimations can be based on quest books and visitor counting by park personnel and local entrepreneurs.

### Social media data

Social media data used in this study includes posts from year 2014 that were collected from Instagram (www.instagram.com/developer), Flickr (www.flickr.com/api) and Twitter (dev.twitter.com/). Overall, Instagram was the most popular social media platform among the three (see Table 1), having the highest number of posts (46%), unique social media users (76%) and social media user days (71%). There are some country and platform specific peculiarities such as the fact that in Finland Twitter had the highest number of posts (52% of all posts). However, the number of unique Twitter users is much lower (16%) compared to Instagram (82%). Flickr is overall the least popular platform with all represented measures.

The data was collected using the Application Programming Interfaces (API) that were provided by the social media platforms (find more details about the data collection system from Hausmann *et al*.^25^). We collected a global database of Instagram, Twitter and Flickr data from national parks (IUCN category II) that are included in the World Database on Protected Areas^61^ covering the years 2014–2016 (temporal coverage varies slightly between platforms). For this study, we selected data only for 2014 because we had fully matching datasets for all three platforms on that year. A spatial query from our global database reveals that 61% (3280/5401) of the national parks in the world had social media activity (in 2014–2016) on the analysed platforms. We selected posts that were within the national parks of South Africa and in Finland using spatial query in PostGIS (based on the coordinates of the posts and park polygons). Only data having a specified location is included (either as geotags or coordinates). Additionally, we used 10 km buffer around the parks to ensure that also posts from water areas in the archipelago (close to islands) were properly included. In Cape Town, South Africa, a small area was excluded from Table Mountain Twitter dataset because it contained all tweets with a place tag “Cape Town” which biased the dataset. Only data that is publicly available was collected.

### Calculating the number of visitors from social media (SUD)

We calculated visitors from social media by first summing the number of active social media users per day (SUD). Thus, if a user uploads five posts on a single day, s/he is counted as one. However, if a user uploads one post on three consecutive days, s/he is counted three times. As the official statistics from the parks were reported on a monthly basis, we aggregated the social media user-days by summing them into a monthly level statistics in order to compare them with official statistics. This approach works as a good surrogate for estimating visitation numbers in the parks that has been acknowledged earlier also by other scientists^18,19^. It is also equivalent to the method how visitors are calculated in Finland national parks using counters: if an individual user is active on the park on multiple days during the visit, s/he is counted multiple times during the visit.

### Statistical methods

We compare the similarity of the park popularity rankings (Fig. 2) between social media data and official statistics by using the Spearman correlation coefficient which is a commonly used statistical method to investigate the monotonic relationship between two variables based on ranks. The relationship in monthly visitation patterns between official visitor statistics and SUD (Figs 3 and 4) were investigated by visualizing the differences in the monthly proportions of visitors per year that standardizes the value scale (possible values between 0.0–1.0) and makes it possible to compare the temporal visitation patterns visually (the sum of monthly proportions equals to 1.0). Additionally, Pearson correlation coefficients were used to measure the linear relationship between observations from the two datasets. We chose to use a simple statistical method (Pearson’s correlation) due to the limited number of observations per park (1 for each month, n = 12) that prevented the use of more sophisticated statistical methods such as regression models designed for count data (could also take into account temporal autocorrelation), or to conduct a Granger causality test that could be used to assess if one time-series could be used to predict the second one.

To assess the differences in the correlation coefficients between different platforms, we visualized boxplots and tested the statistical significance of the differences of the park-wise correlation coefficients (Fig. 5) with Kruskal-Wallis and Dunn’s tests. We chose a Kruskall-Wallis test which is a non-parametric version of ANOVA since our data did not meet the assumption of homoscedasticity (prerequisite for ANOVA), as the standard deviations of the groups were not all equal. Furthermore, we used Dunn’s post hoc test to assess whether there were significant differences between individual social media platforms (null-hypothesis: no difference between groups). The reported p-values were adjusted according to the Holm-Sidak measure that was used to control for family-wise error rate. Holm-Sidak measure was chosen instead of a more conservative Bonferroni measure, as we can assume that the measurements between the groups are independent and there are no ties between the groups.

Finally, we assessed the relationship between the Pearson correlations and the popularity of the park (based on log transformed visitor count and social media user days) by visualizing them with a scatter plot combined with fitted trend line in Fig. 6. We also present the Pearson correlation coefficients to inform about the linear relationship between the variables and the slope from the fitted linear trend line.

As visitor statistics and SUD are both time-series data, there might exist temporal autocorrelation in the datasets that might affect the Pearson correlation coefficients. Hence, we investigated the datasets for possible temporal autocorrelation by visualizing the correlograms using Autocorrelation Function (ACF) and Partial Autocorrelation Function (PACF) with different lags (up to 11 lags) that revealed if the measurements from different months in the parks are autocorrelated (see Supplement S1 for details). Those parks with temporal autocorrelation were removed from the analyses presented in Figs 5 and 6.

We share all the codes that were used to analyse the data, and produce and visualize the results presented in this paper in GitHub (www.github.com/DigitalGeographyLab/ASOMERE).

### Assessment of the temporal patterns and usability of social media data with stakeholders

We assessed the results of this study by three semi-structured group-interviews in order to understand the local factors that explain differences and similarities in the comparisons. We went through the results park by park with experts (n = 22) consisting of park managers, visitor statistics coordinators, and communications officers from the national park authorities. Two workshops were organized for SANParks authorities in South Africa at Kruger National Park in Skukuza (March 11^th^ 2016) and in the city of Knysna (March 15^th^ 2016) and one workshop for authorities of Metsähallitus in Helsinki, Finland (October 26^th^ 2016). During the workshops, managers provided feedback on the results and provided suggestions of factors that could explain both converging and diverging patterns between social media and official statistics.

### Data availability

Relevant tools and data related to the article (restricted by terms of service of social media platforms) can be found from: www.github.com/DigitalGeographyLab/ASOMERE.

## Electronic supplementary material

Supplementary Information
