# Supplementary material for: Instagram, Flickr, or Twitter: Assessing the usability of social media data for visitor monitoring in protected areas
Source: Sci Rep. 2017 Dec 14;7:17615. doi: 10.1038/s41598-017-18007-4 (PMC5730565; doi:10.1038/s41598-017-18007-4)
Supplement: Supplementary file 1 — Supplementary Information [file 41598_2017_18007_MOESM1_ESM.pdf]

Supplement associated with article:

Instagram, Flickr, or Twitter: Assessing the usability of social media data for visitor monitoring in protected areas (Scientific Reports)

Henrikki Tenkanen, Enrico Di Minin, Vuokko Heikinheimo, Anna Hausmann, Marna Herbst, Liisa Kajala & Tuuli Toivonen

Contents

S1. Estimating the temporal autocorrelation in the data 1
S2. Platform comparisons including all parks 9
S3. Potential reasons for difference between official statistics and social media data 11
References 13

S1. Estimating the temporal autocorrelation in the data

Pearson correlation coefficient is a widely used statistical measure to study the linear relationship between two variables, i.e. if the variables are correlated with each other. In case of time-series data, however, Pearson correlation estimates might be inflated (i.e. showing stronger relationship than there actually is) because of temporal autocorrelation on the measurements that have been done on the same site on regular intervals. Because our data contains monthly measurements from the same locations (national parks), there is a change of having temporal autocorrelation in the data that could potentially influence the correlations. Hence, we used autocorrelation function (ACF) and partial autocorrelation function (PACF) to estimate the temporal autocorrelation in the datasets (official visitor statistics and social media user days (SUD)). We plotted the correlograms (autocorrelation plots) for each park in South Africa (Figure S1) and Finland (Figure S2) with different lags (up to 11 lags) using 95 % confidence interval. If the autocorrelation is higher / lower than the confidence limits (i.e. outside the blue area in the Figures S1 and S2), there exists temporal autocorrelation in the data. In such cases, the Pearson correlation coefficient should be taken with caution as the correlation might be inflated. We removed such parks from the further analyses (Figures 5 and 6 in the main article).

The correlograms show that in some of the parks there is temporal autocorrelation within the data. In South Africa 3/21 parks had slight temporal autocorrelation (with 95 % confidence interval): the official visitor statistics from Camdeboo and Tsitsikamma national parks had temporal autocorrelation with lag 1, whereas the social media data from Mokala national park had temporal autocorrelation with lag 2 (see Figure S1). In Finland, altogether 17/35 of the national parks had temporal autocorrelation: 6/35 of the national parks (Helvetinjärvi, Isojärvi, Nuuksio, Oulanka, Repovesi, Seitseminen) had temporal autocorrelation in SUD, and 16/35 in official visitor statistics (Helvetinjärvi, Isojärvi, Lauhanvuori, Leivonmäki, Lemmenjoki, Liesjärvi, Nuuksio, Patvinsuo, Petkeljärvi, Pyhä-Häkki, Päijänne, Repovesi, Rokua, Salamajärvi, Seitseminen, Selkämeri). Most of the parks had temporal autocorrelation with 1 or 2 lags.

1A

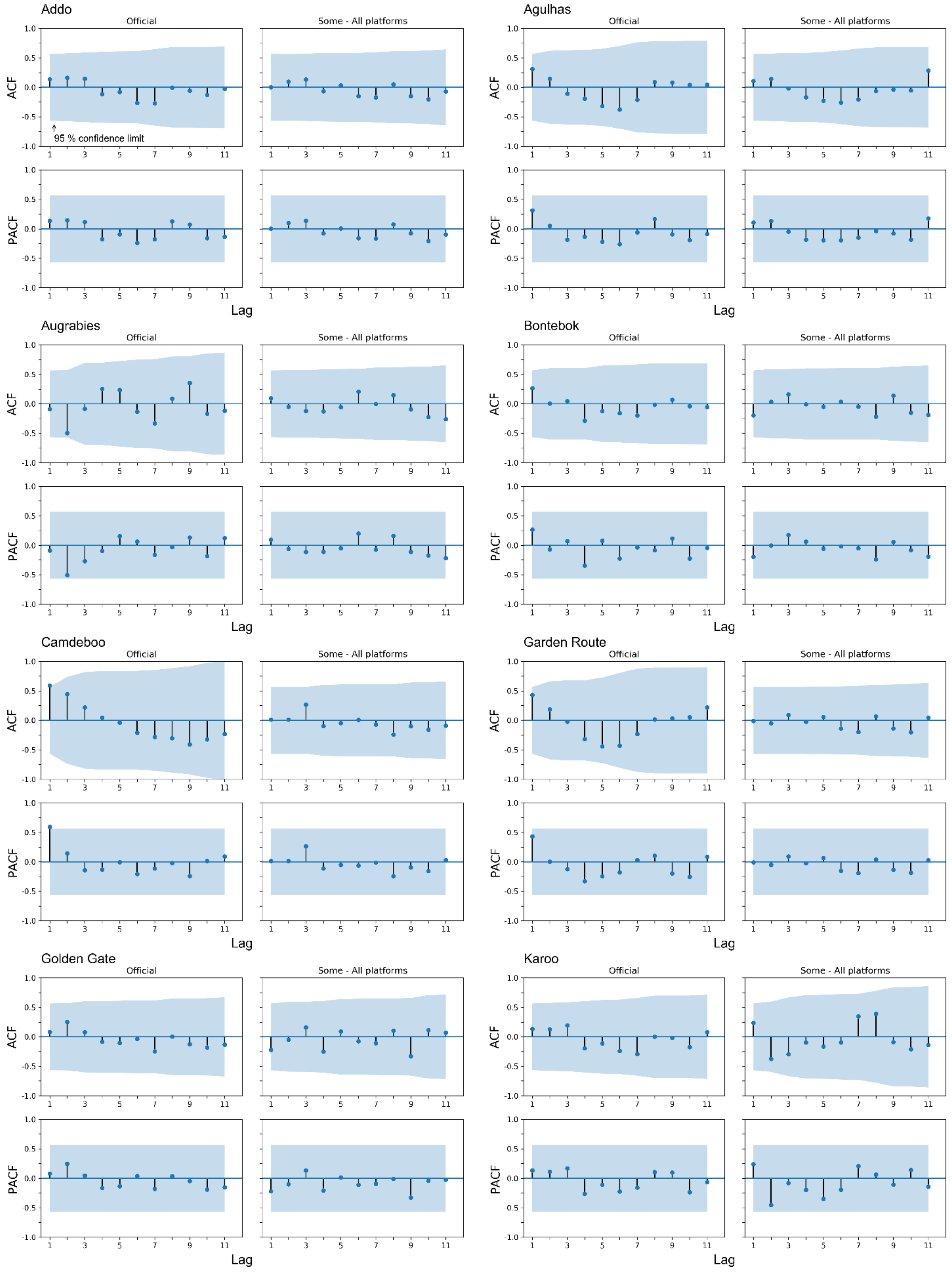

Addo
Official
Some - All platforms
95 % confidence limit
ACF
PACF
Lag
Agulhas
Augrabies
Bontebok
Camdeboo
Garden Route
Golden Gate
Karoo

1B

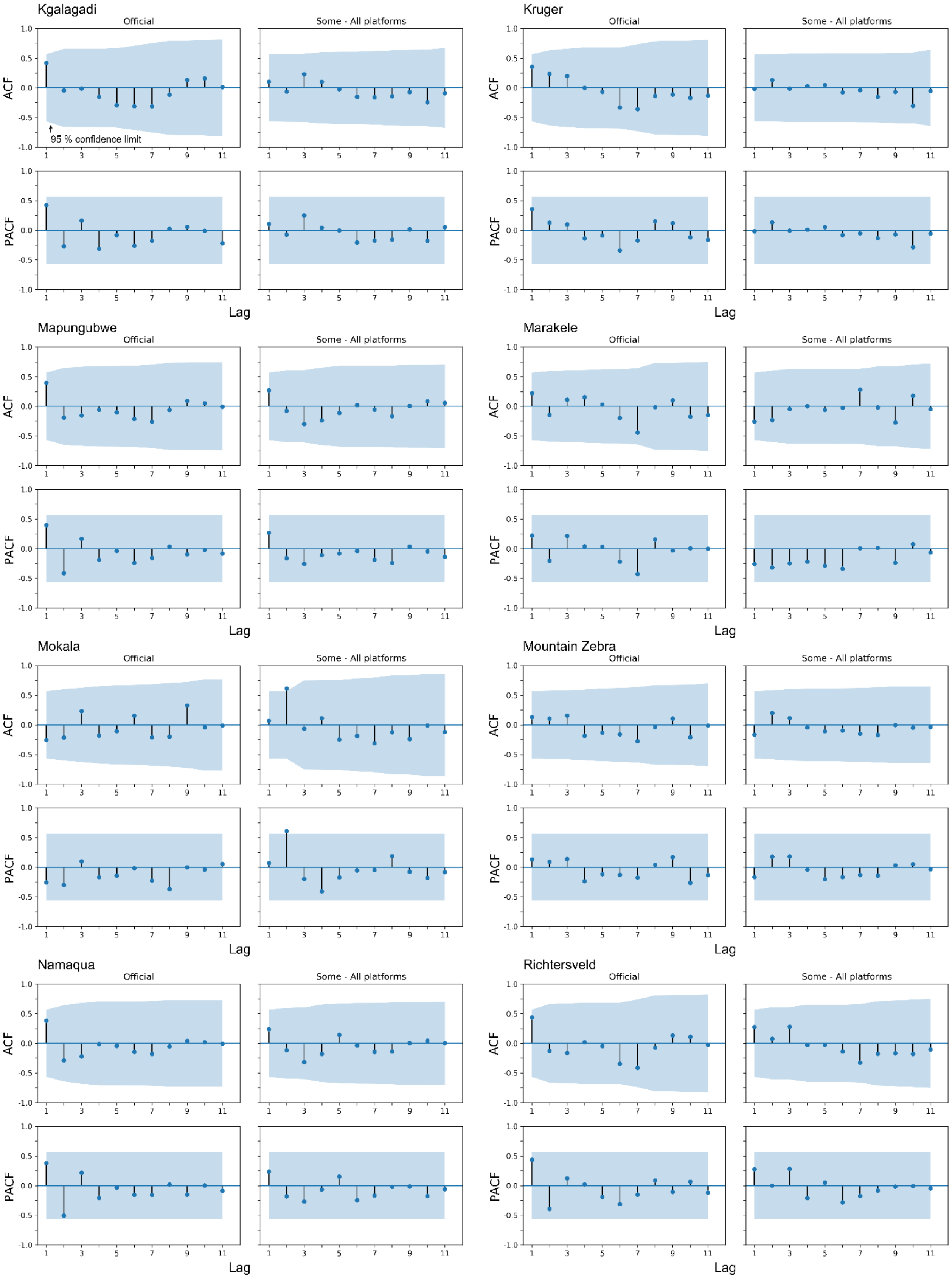
Kgalagadi
Official
Some - All platforms
ACF
PACF
95 % confidence limit
Lag
Kruger
Mapungubwe
Marakele
Mokala
Mountain Zebra
Namaqua
Richtersveld

1C

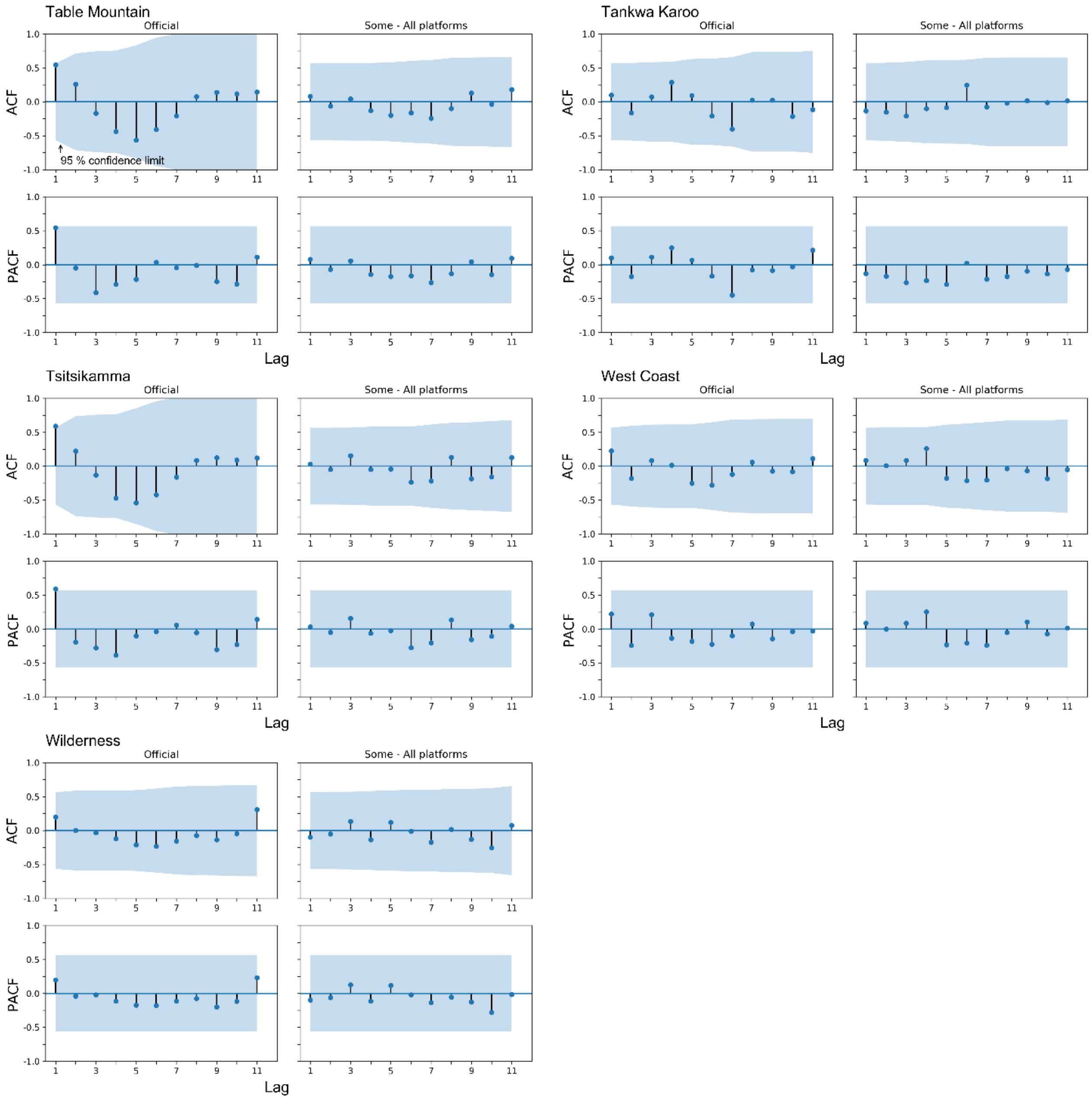

Figure S1. *Correlograms (1A-1C) for South African national parks showing the (temporal) autocorrelation function (ACF) and partial autocorrelation function (PACF).*

2A

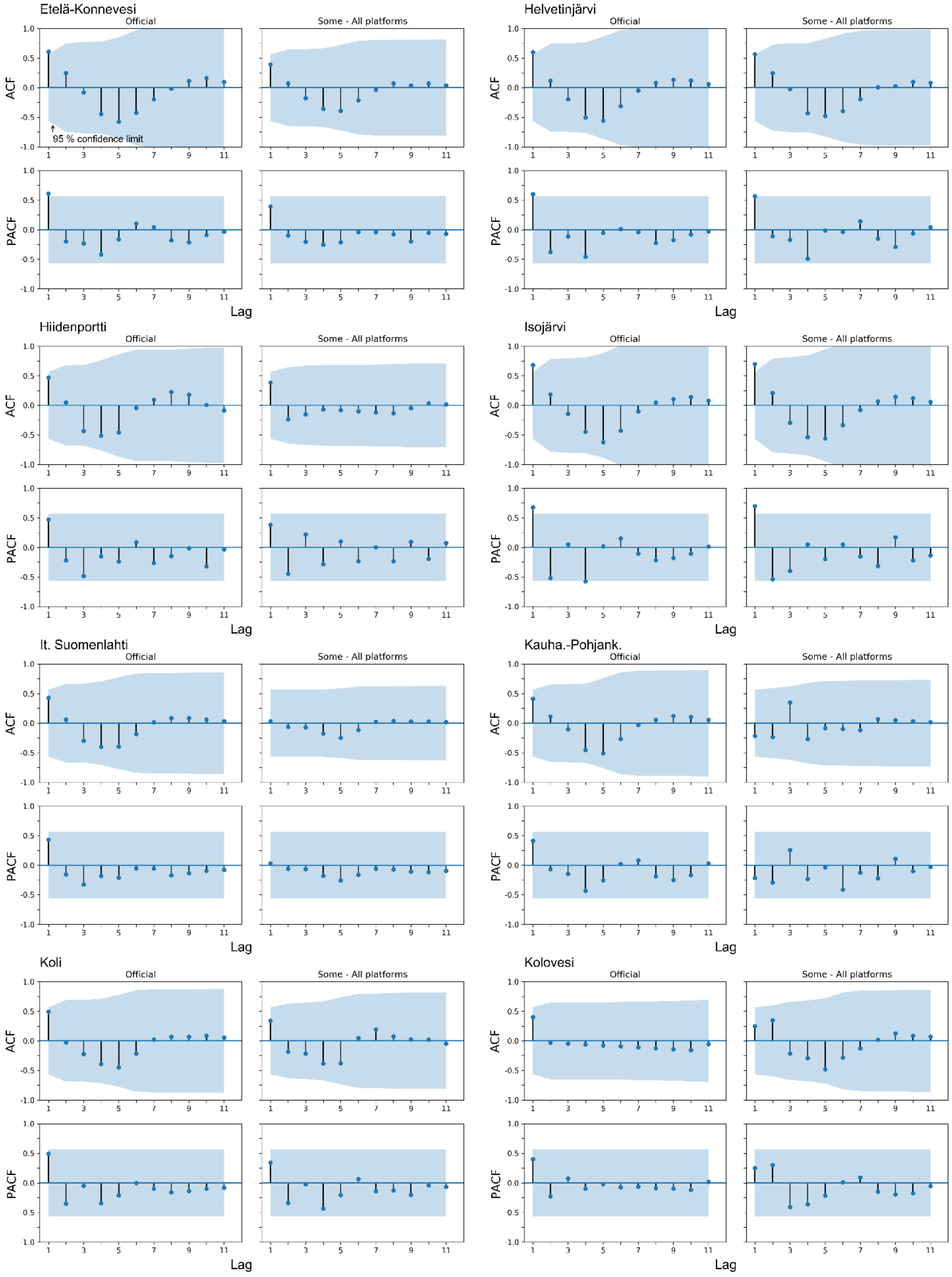
Etelä-Konnevesi
Helvetinjärvi
Hiidenportti
Isojärvi
It. Suomenlahti
Kauha.-Pohjank.
Koli
Kolovesi
Official
Some - All platforms
ACF
PACF
Lag
95 % confidence limit

2B

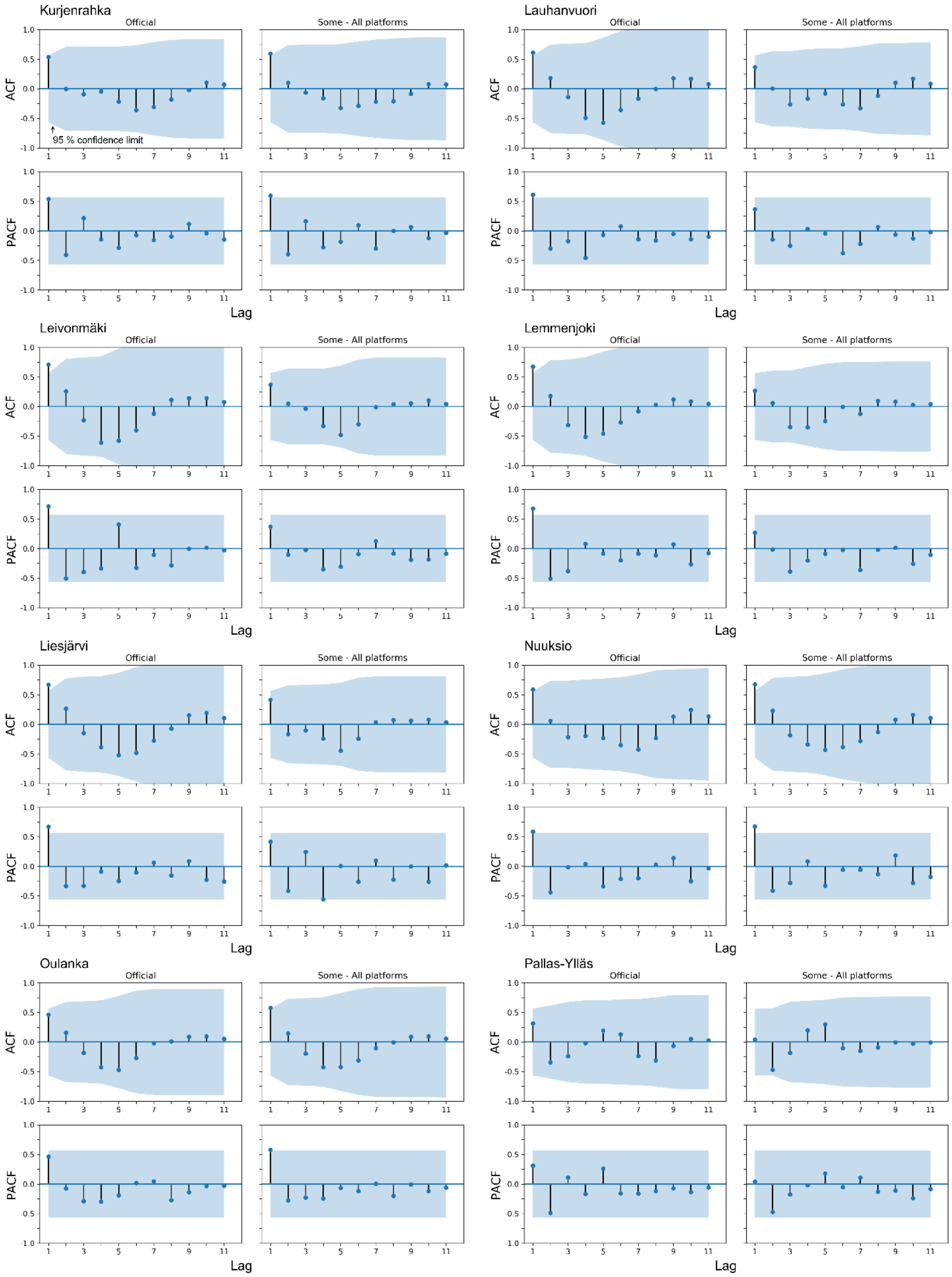
Kurjenrahka
Lauhanvuori
Leivonmäki
Lemmenjoki
Liesjärvi
Nuuksio
Oulanka
Pallas-Ylläs
Official
Some - All platforms
ACF
PACF
Lag
95 % confidence limit

2C

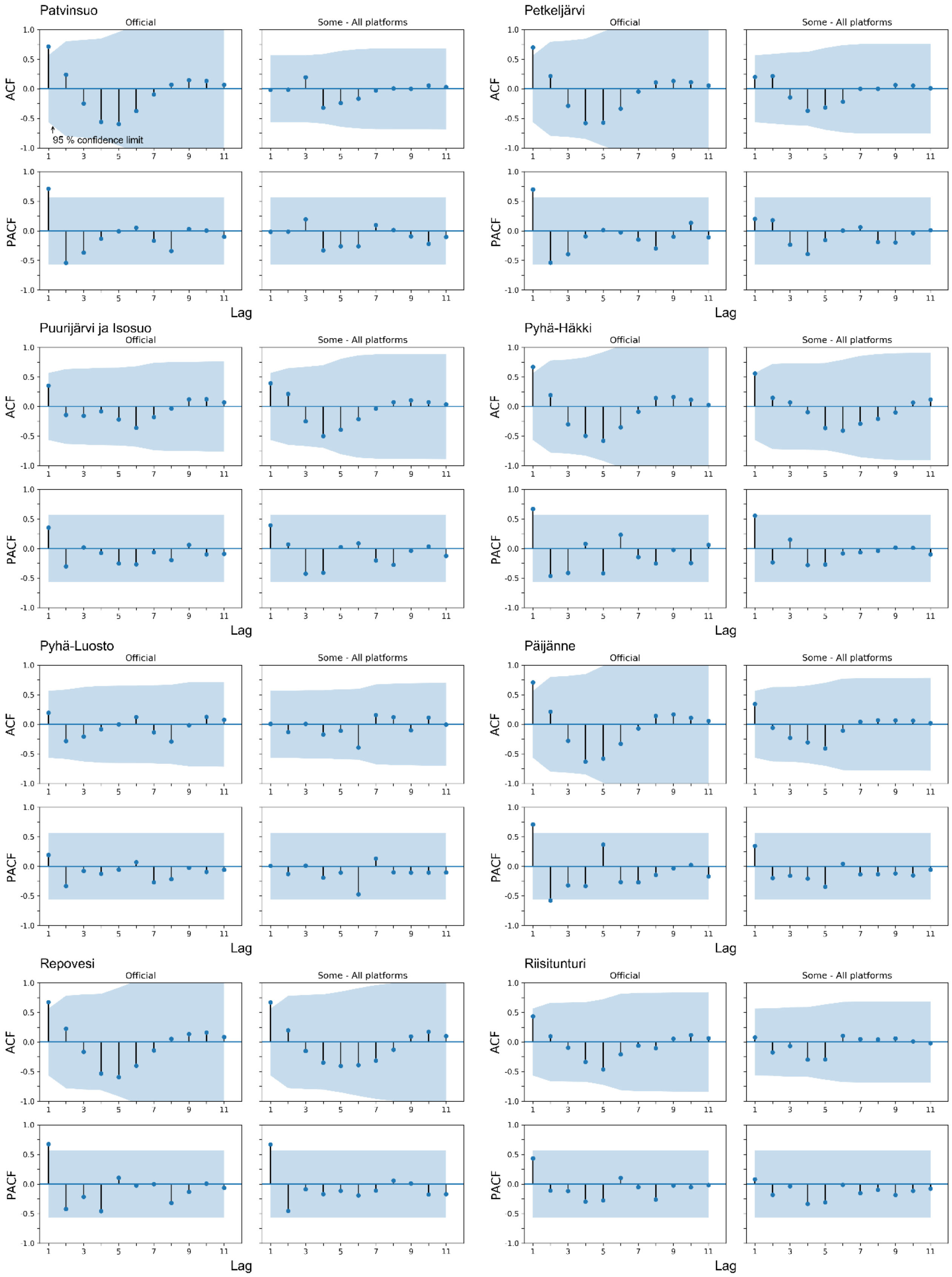
Patvinsuo
Official
Some - All platforms
ACF
PACF
95 % confidence limit
Lag
Petkeljärvi
Official
Some - All platforms
ACF
PACF
Lag
Puurijärvi ja Isosuo
Official
Some - All platforms
ACF
PACF
Lag
Pyhä-Häkki
Official
Some - All platforms
ACF
PACF
Lag
Pyhä-Luosto
Official
Some - All platforms
ACF
PACF
Lag
Päijänne
Official
Some - All platforms
ACF
PACF
Lag
Repovesi
Official
Some - All platforms
ACF
PACF
Lag
Riisitunturi
Official
Some - All platforms
ACF
PACF
Lag

2D

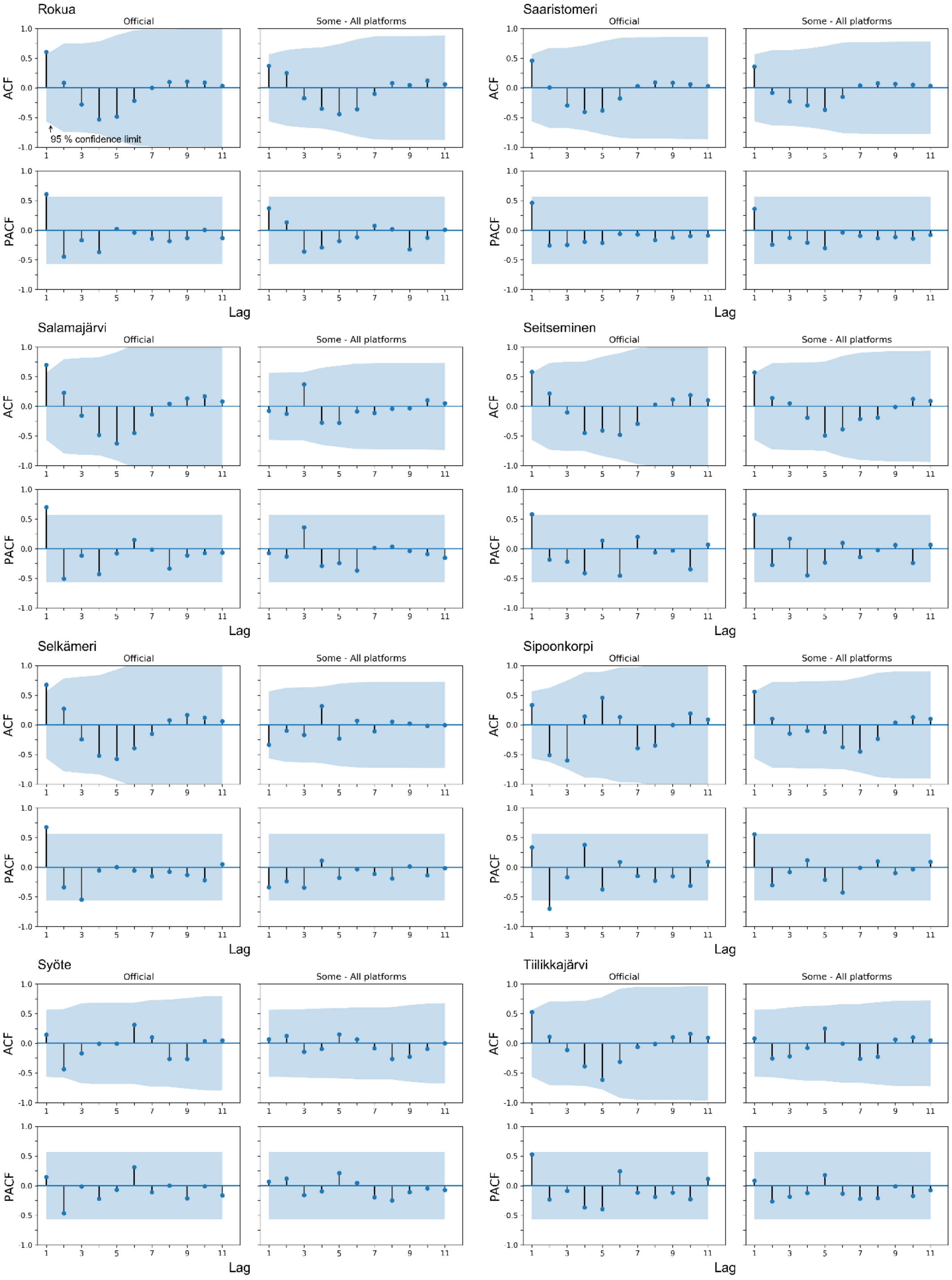
Rokua
Official
Some - All platforms
95 % confidence limit
Saaristomeri
Salamajärvi
Seitseminen
Selkämeri
Sipoonkorpi
Syöte
Tiilikkajärvi
ACF
PACF
Lag

2E

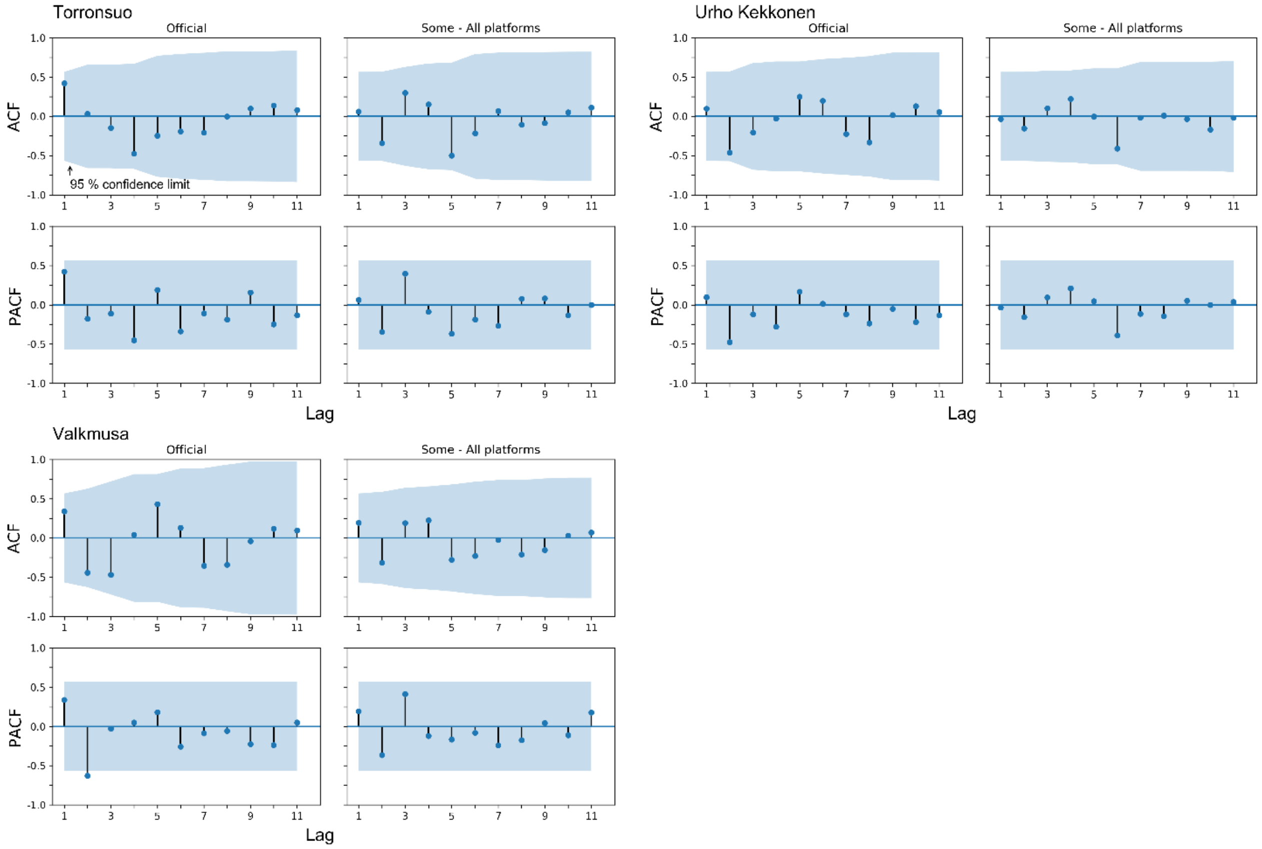

Figure S2. *Correlograms (2A-2E) for Finnish national parks showing the (temporal) autocorrelation function (ACF) and partial autocorrelation function (PACF).*

S2. Platform comparisons including all parks

In the main text of the article we exclude parks with temporal autocorrelation that reduced the number of parks included in the analyses in South Africa from 21 to 18 and in Finland from 35 to 18 (altogether n=36). In South Africa, where only a few parks had temporal autocorrelation, this exclusion did not have a dramatic effect on the results. However, in Finland where almost half of the parks were excluded, the platform comparisons produced slightly different results. Hence, in Figures S3 and S4 we report also the results including all national parks in Finland and South Africa.

Results in Figure S3 show that the differences between platforms are statistically significant also in Finland with p-value 0.008 when tested with Kruskal-Wallis test. Dunn's test with Holm-Sidak adjusted p-values reveal that the differences between Instagram and Twitter (p-value: 0.008) and Instagram and Flickr (p-value: 0.009) are both significant. With all parks included, interestingly, the

median correlations of Instagram and Twitter are the same in both countries (platform-wise) which might indicate the general robustness of social media for predicting the visitor rates in an equivalent manner in very different and distinct regions (here South Africa vs Finland). However, these results might be biased because of temporal autocorrelation. It is also noteworthy that Flickr does not follow the same pattern as it has clearly better correlations in Finland than it has in South Africa.

Figure S4 focuses on investigating if social media data tend to work better in more visited parks and in parks with more social media content and users. The results reveal that the correlations of Instagram are clustered around 0.75 correlation coefficient, whereas with Twitter and Flickr the correlations are more scattered when including all national parks. When considering all national parks, the trendline does not have as strong slope as in Figure 6 (main text) where only parks without temporal autocorrelation were reported.

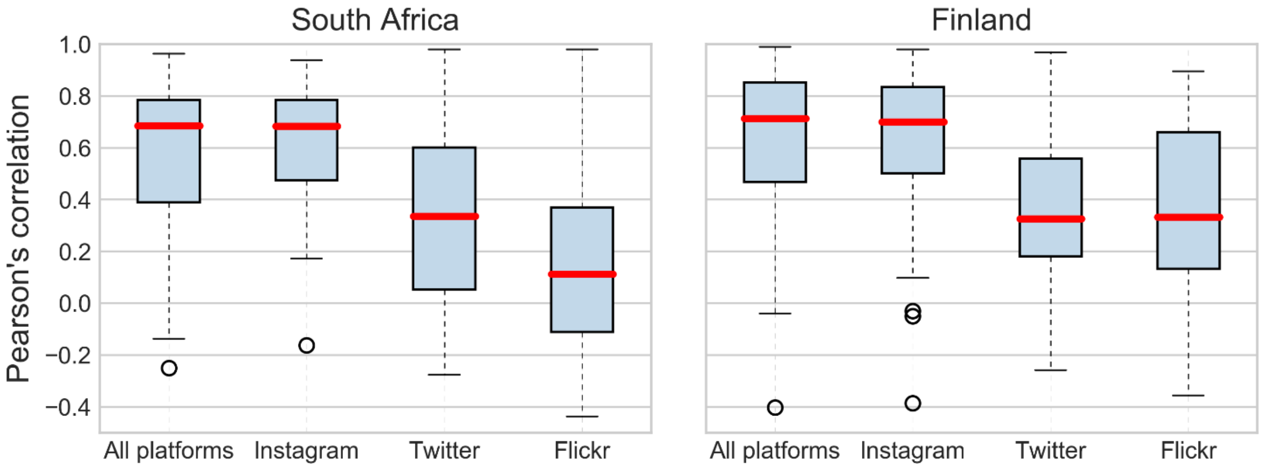

Figure S3. *Boxplots including all national parks (n=56) reveal that Instagram performs best in estimating the monthly visitors when measured with Pearson's correlation coefficients between official visitor statistics and social media user-days (see also Figure 5 in the article). The performance of Instagram is rather similar both in South Africa and in Finland, having a 70 % median correlation. Figure has been created with Matplotlib v2.02* [1] *and Pandas v0.19.2 modules in Python 3.5.3 programming language (www.python.org/) under the PSF License (docs.python.org/3/license.html).*

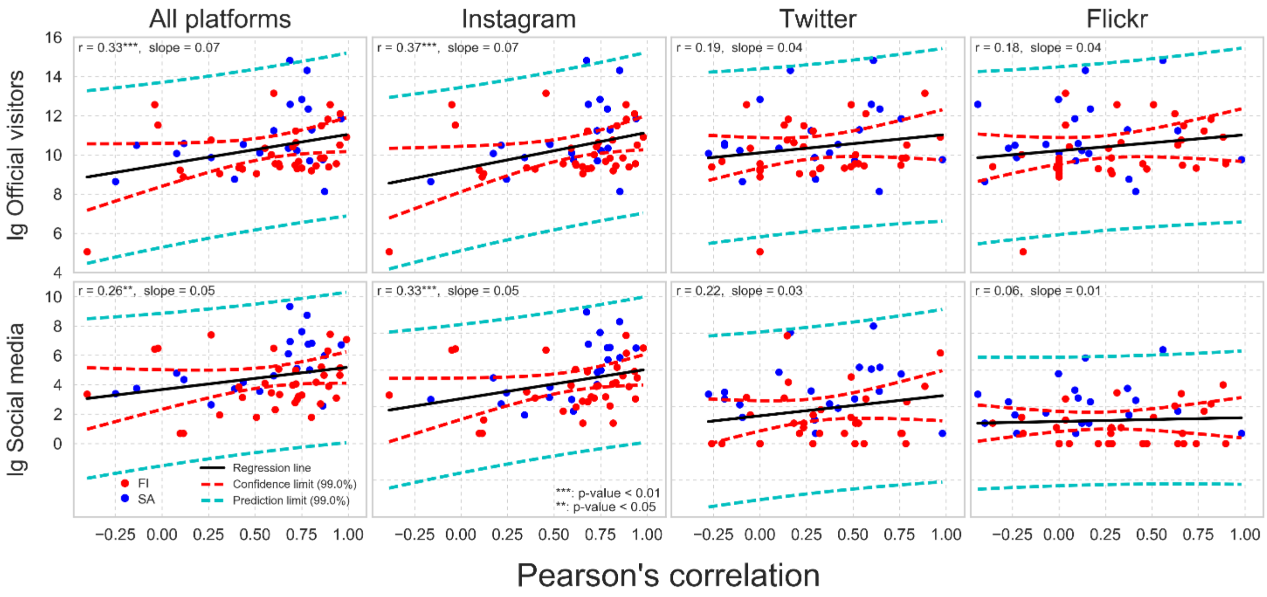

Figure S4. *Scatter plots with trend lines including all 56 national parks in South Africa and Finland (see also Figure 6 in the article). Social media data tend to work better in more visited parks (top row) and in parks having higher number of social media user days (bottom row) which is revealed by comparing the Pearson correlation coefficients between monthly visitation numbers between social media and official visitor statistics against log-transformed number of official visitors and social media user-days. Figure has been created with Matplotlib v2.02* [1] *and Pandas v0.19.2 modules in Python 3.5 programming language (https://www.python.org/) under the PSF License (docs.python.org/3/license.html).*

S3. Potential reasons for difference between official statistics and social media data

In the group discussions in South Africa and Finland, the stakeholders identified hypothetical reasons which could influence the differences between social media posts and visitor numbers. We organised these potential reasons presented in the group discussions under four main categories (Figure 7 in the article). Below and in Figure S5, we provide some concrete examples taken from the stakeholder discussions.

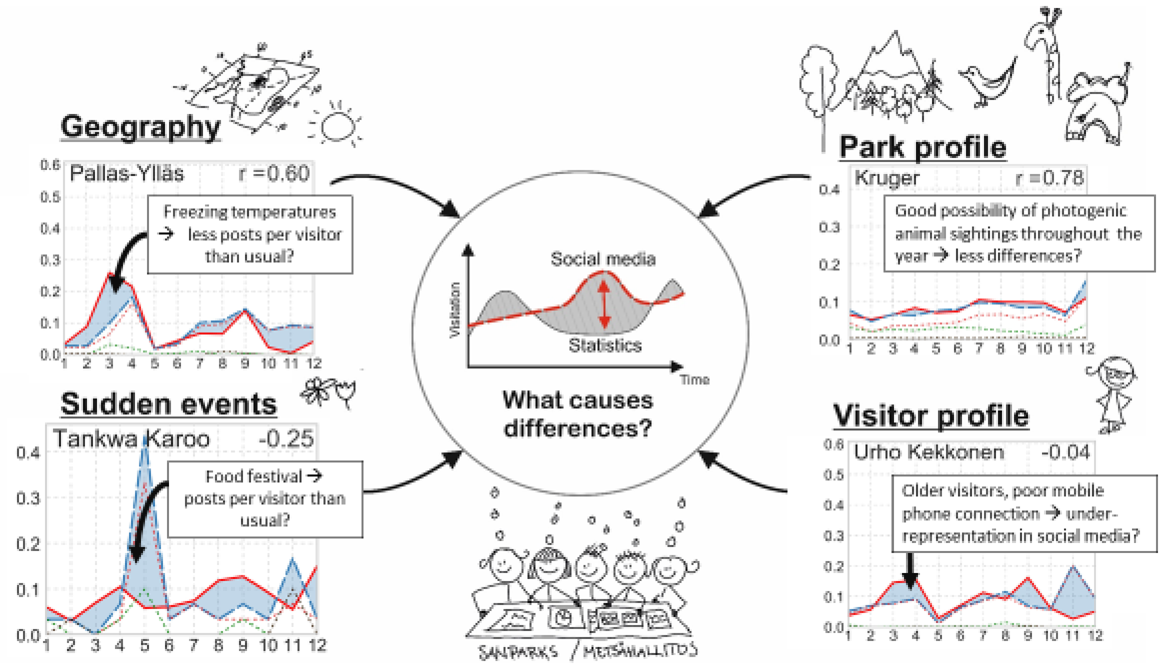

Figure S5. *Examples of discrepancies between the social media user days (SUD) and the official statistics in four different parks, as hypothesised by the park personnel in the stakeholder workshops. The examples are chosen to match the four broad categories of explanatory factors, as presented in Fig 7 of the main text.*

Firstly, geographical location of parks may influence social media posting behaviour. For example, in parks located in high latitudes, climatic conditions (e.g. coldness and the amount of light hours in winter) can influence visitors' willingness to take photos in outdoor conditions. For example, the drop in social media activity (compared to the respective statistics) in Pallas-Yllästunturi national park in the Finnish Lapland in winter months might be caused by cold weather that influences the use of smart phones outdoors.

Secondly, the profile of the park may influence activity of people in social media. If the park is popular and provides equally photogenic events or activities throughout the year, it is likely that the social media activity matches well the visitor statistics. A good example is Kruger National park in South Africa, where visitors are likely to see iconic African animals throughout the year. Hence in Kruger, the visitor statistics and the social media activity matches relatively well.

Thirdly, the profile of visitors (age, gender etc.) may influence the use of social media during park visits and the availability of data. For instance, the poor relationship between social media posts and official visitors' statistics of some of the parks in Finland (e.g. Urho Kekkonen) might be related to the older average age of the visitors who are less active on social media: the average age in 2010 was 44 in Urho Kekkonen (2nd most visited park in Finland) whereas e.g. in Nuuksio (3rd most visited park) it was only 38.

Finally, social and phenological events may affect the relative number of social media posts. For example, festivals can cause peaks in the temporal pattern of social media data (e.g. Tankwa Karoo). Moreover, rains and poor weather conditions may discourage people to take photos, while a particularly beautiful spring flower blooming may attract more tourists to parks and increase their willingness to post.

The discussion above is based on expert knowledge and reasoning in the stakeholder workshops. These reasons should be investigated with more thorough analyses in future studies of the factors influencing the use of social media in the parks.

References

1. Hunter, J. D. Matplotlib: A 2D Graphics Environment. *Comput. Sci. Eng.* **9,** 90–95 (2007).
